# Tyrosine phosphorylation of WIP releases bound WASP and impairs podosome assembly in macrophages

**DOI:** 10.1242/jcs.154880

**Published:** 2015-01-15

**Authors:** Vineetha Vijayakumar, James Monypenny, Xing Judy Chen, Laura M. Machesky, Sergio Lilla, Adrian J. Thrasher, Inés M. Antón, Yolanda Calle, Gareth E. Jones

**Affiliations:** 1 Randall Division of Cell and Molecular Biophysics, King’s College London, London SE1 1UL, UK; 2 The Beatson Institute for Cancer Research, Glasgow G61 1BD, UK; 3 Section of Molecular and Cellular Immunology, Institute of Child Health, University College London, London WC1N 1EH, UK; 4 Centro Nacional de Biotecnología (CNB-CSIC), 28049 Madrid, Spain; 5 Department of Haematological & Molecular Medicine, King's College London, London SE5 9NU, UK

**Keywords:** WIP, WIPF1, Tyrosine phosphorylation, Podosome, Actin cytoskeleton, Macrophages, Matrix degradation

## Abstract

Podosomes are integrin-containing adhesion structures commonly found in migrating leukocytes of the monocytic lineage. The actin cytoskeletal organisation of podosomes is based on a WASP- and Arp2/3-mediated mechanism. WASP also associates with a second protein, WIP (also known as WIPF1), and they co-localise in podosome cores. Here, we report for the first time that WIP can be phosphorylated on tyrosine residues and that tyrosine phosphorylation of WIP is a trigger for release of WASP from the WIP–WASP complex. Using a knockdown approach together with expression of WIP phosphomimics, we show that in the absence of WIP–WASP binding, cellular WASP is rapidly degraded, leading to disruption of podosomes and a failure of cells to degrade an underlying matrix. In the absence of tyrosine phosphorylation, the WIP–WASP complex remains intact and podosome lifetimes are extended. A screen of candidate kinases and inhibitor-based assays identified Bruton's tyrosine kinase (Btk) as a regulator of WIP tyrosine phosphorylation. We conclude that tyrosine phosphorylation of WIP is a crucial regulator of WASP stability and function as an actin-nucleation-promoting factor.

## INTRODUCTION

Adhesion and migration of monocyte-derived cells is regulated by actin-rich integrin-containing structures termed podosomes (Calle et al., 2006; Linder and Aepfelbacher, 2003). The rapid formation and turnover of podosomes during migration is dependent upon the actions of the Wiskott–Aldrich syndrome protein (WASP) (Calle et al., 2004b; Calle et al., 2004a), a key regulator of Arp2/3-dependent *de novo* actin polymerisation (Millard et al., 2004). In cells, WASP is associated with the WASP-interacting protein (WIP, also known as WIPF1) (Stewart et al., 1999; Ramesh et al., 1997), a multifunctional adaptor implicated in a wide range of cellular functions, including cell adhesion, migration and chemotaxis, T-cell activation and proliferation, and intracellular pathogen motility (Anton and Jones, 2006; Antón et al., 2007; Moreau et al., 2000). WIP functions through binding to both globular and filamentous actin (Martinez-Quiles et al., 2001) and several regulators of actin dynamics (Antón et al., 1998). WIP can also bind to and regulate the function of the actin-nucleation-promoting factor cortactin (Kinley et al., 2003; Bañón-Rodríguez et al., 2011). In cells of haematopoietic origin, WIP is an important regulator of WASP, the expression of which is restricted to cells of this lineage. WASP is indispensable for normal leukocyte function and its importance is highlighted in the congenital disorder Wiskott–Aldrich syndrome in which missense mutations in the *WAS* gene result in severe immunodeficiency (Derry et al., 1994; Ochs and Thrasher, 2006; Thrasher and Burns, 2010).

WIP regulates WASP expression levels by binding to and protecting WASP from calpain- and/or proteasome-mediated degradation (Blundell et al., 2009; Chou et al., 2006; de la Fuente et al., 2007; Macpherson et al., 2012). Under resting conditions, the majority of WASP forms a complex with WIP, and any unbound WASP is rapidly targeted for degradation (Tsuboi, 2007; Konno et al., 2007; Macpherson et al., 2012). Given the crucial role of WASP in immune cell function, it is unsurprising that mutations in WASP which impair or abolish WIP binding result in immunological disorders of varying severity (Kim et al., 2004; Stewart et al., 1999). WIP-null mouse dendritic cells exhibit defects in polarity, chemotaxis and cytoskeletal organisation (Bañón-Rodríguez et al., 2011; Chou et al., 2006), phenotypes reminiscent of those found for WASP-null dendritic cells (Burns et al., 2001; Calle et al., 2004a) and macrophages (Jones et al., 2002; Zicha et al., 1998). Importantly, WIP and WASP are essential for the assembly and turnover of podosomes, actin-rich adhesions implicated in the invasion and matrix remodelling of professional migratory cells such as macrophages, dendritic cells and osteoclasts (Calle et al., 2004b; Chabadel et al., 2007). Macrophages and dendritic cells from WAS patients fail to form podosomes and this is likely to be a major contributing factor to the defective trafficking and immune surveillance of these cells that are characteristic of this disease (Bouma et al., 2009; Burns et al., 2004; Jones et al., 2002; Thrasher, 2002).

Although the ability of WIP to protect WASP from proteolytic degradation is vital for WASP function in podosome formation, WIP has also been shown to contribute directly to the regulation of these structures, targeting WASP to sites of podosome assembly (Chou et al., 2006). Mechanisms that control WIP–WASP interaction are therefore crucial for the regulation of podosome function and consequently normal leukocyte biology as they influence both WASP localisation and turnover. However, the nature of the regulatory mechanisms that control WIP function has remained elusive. Phosphorylation represents a strong candidate for regulation of WIP function, as studies have reported serine/threonine phosphorylation of WIP on a number of residues (Dong et al., 2007; Krzewski et al., 2006; Sasahara et al., 2002; Shu et al., 2004). Of these, only S488 had been the basis of any functional study (Dong et al., 2007; Krzewski et al., 2006; Sasahara et al., 2002), it being reported to be phosphorylated in a PKCθ-dependent manner in response to T-cell receptor activation (Sasahara et al., 2002). S488 lies immediately downstream of the WASP-binding domain (WBD) of WIP (amino acids 451–485) (Volkman et al., 2002; Zettl and Way, 2002) and it was originally proposed that phosphorylation of this residue results in dissociation of the WIP–WASP complex (Sasahara et al., 2002). However, subsequent studies in both Jurkat cells (Dong et al., 2007) and the YTS natural killer cell line (Krzewski et al., 2006) have demonstrated that WIP and WASP can remain bound together following phosphorylation of this residue, and the functional significance of this phosphorylation event has only recently been addressed (Fried et al., 2014).

In this study, we provide evidence for tyrosine phosphorylation of WIP as a mechanism for disrupting WIP–WASP association. We demonstrate that phosphomimetic but not unphosphorylatable mutations of tyrosine residues within the WBD of WIP abolish WASP binding, fail to protect WASP from degradation, and are defective in their ability to rescue podosome formation and their matrix degradation properties when expressed in WIP-knockdown cells. Conversely, expression of wild-type and unphosphorylatable mutants in WIP-knockdown cells results in the restoration of podosomes and matrix degradation. Unequivocal confirmation of the phosphorylation of tyrosine residues within the WBD of WIP was provided by mass spectroscopy. A screen of candidate tyrosine kinases and subsequent small molecule inhibitor and kinase assays identified Bruton's tyrosine kinase (Btk) as an effector of WIP tyrosine phosphorylation. Taken together, our findings provide evidence for tyrosine phosphorylation of WIP as a mechanism for regulating WIP–WASP association and podosome function in cells of the monocytic lineage.

## RESULTS

### Putative serine/threonine phosphorylation sites in WIP do not regulate WIP–WASP association

**Fig. 1.**
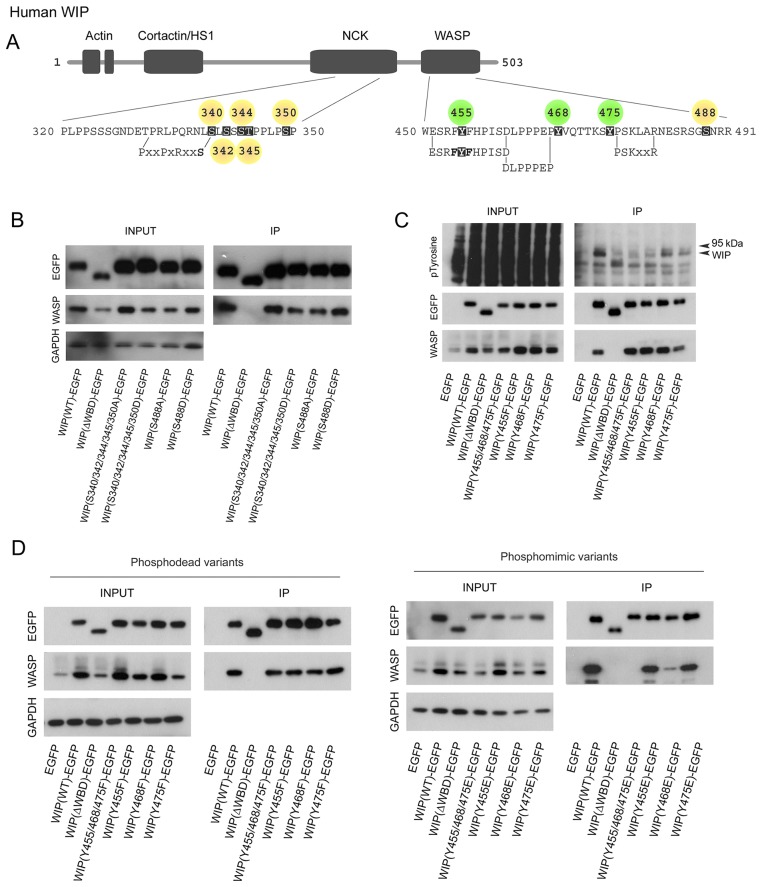
**Evidence of tyrosine phosphorylation of WIP.** (A) Schematic of human WIP depicting both known and putative phosphorylation sites and their position with respect to functional domains. Amino acid sequences representing regions of functional importance are also shown, highlighting the proximity of respective phosphorylation sites. Serine/threonine residues are in yellow and tyrosine residues in green. (B) Western blots from immunoprecipitation assays examining the importance of putative WIP serine/threonine phosphorylation sites in determining WASP binding. WIP–EGFP variants encoding serine/threonine phosphomimetic (serine to aspartic acid) and unphosphorylatable mutations (serine to alanine) were expressed in THP-1 cells by using lentiviral gene transduction. The exogenously expressed protein was subsequently immunoprecipitated (IP) with anti-GFP antibody to determine the amount of bound endogenous WASP. Wild-type WIP and WIP lacking the C-terminal WBD (ΔWBD) were included as positive and negative controls, respectively. (C) Western blots from immunoprecipitation assays to determine whether the tyrosine residues of WIP represent targets for phosphorylation. WIP–EGFP fusion proteins with all or individual tyrosine residues replaced with the unphosphorylatable mimic phenylalanine were immunoprecipitated from THP-1 lysates following pretreatment of cells with pervanadate. The immunoprecipitated wild-type and unphosphorylatable variants of WIP were then probed for tyrosine phosphorylation by western blotting. (D) Western blots from immunoprecipitation assays examining the importance of WIP tyrosine residues in determining WASP binding. WIP–EGFP fusions harbouring unphosphorylatable (left panel) and phosphomimetic (right panel) tyrosine mutations were immunoprecipitated from THP-1 lysates and WASP binding subsequently assessed as in B.

A number of studies have reported serine/threonine phosphorylation of WIP at residues that lie either within or adjacent to the NCK- and WASP-binding domains (Fig. 1A, yellow residues). NCK is an ubiquitous adaptor molecule known to contribute towards N-WASP–WASP activation and release of its auto-inhibited conformation (Rohatgi et al., 2001). PKCθ-dependent phosphorylation of S488 of WIP in response to T-cell receptor activation has been demonstrated in Jurkat T-cells (Dong et al., 2007), but increasing evidence indicates that this is not associated with disruption of the WIP–WASP complex as previously proposed (Sasahara et al., 2002). In the monocytic THP-1 cell line, we demonstrate that WASP is bound to both unphosphorylatable and phosphomimetic variants of S488 suggesting that phosphorylation of this residue is not associated with the dissociation of the WIP–WASP complex in monocytic cells (Fig. 1B). We also set out to test the ability of other previously suspected phosphorylatable serine/threonine residues. The expression of two WIP constructs that confer either unphosphorylatable or phosphomimetic mutations for five serine/threonine residues (S340, S342, S344, T345 and S350) previously identified as phosphorylated in proteomic screens (Shu et al., 2004) were also able to immunoprecipitate WASP. These results suggest that serine/threonine phosphorylation of WIP, and in particular phosphorylation of S488, which lies close to the WBD of WIP, does not disrupt WIP–WASP interaction.

### Evidence for phosphorylation of tyrosine residues within the WBD of WIP in THP-1 cells

Although serine phosphorylation of WIP has been reported, nothing is currently known about the potential role of tyrosine phosphorylation in the regulation of WIP function. Human WIP contains three tyrosine residues, Y455, Y468 and Y475, which lie in close proximity to one another either within or adjacent to functionally important regions within the WBD of WIP (Fig. 1A, green residues).

To determine whether WIP is tyrosine phosphorylated, EGFP-tagged versions of wild-type WIP [WIP(WT)], the unphosphorylatable WIP mutants Y455F, Y468F and Y475F, and as the triple unphosphorylatable mutant Y455F/Y468F/Y475F (Y455/468/475F) were stably expressed in THP-1 cells following lentiviral transduction. As negative controls, cells expressing either EGFP or a WIP truncation mutant lacking residues 450–503 [WIP(ΔWBD)–EGFP], comprising the WBD and therefore all tyrosine residues, were also included. EGFP fusion proteins were immunoprecipitated from lysates of TGFβ1-differentiated THP-1 cells pre-treated with pervanadate using an anti-GFP antibody. A phosphotyrosine band corresponding to the size of the WIP–EGFP fusion protein was clearly observed for cells expressing the wild-type protein (Fig. 1C). However, the intensity of this band was greatly diminished for cells expressing either the Y455/468/475F triple unphosphorylatable mutant or the single Y455F mutant, and was of similar intensity to that of the WIP(ΔWBD)–EGFP negative control. The intensity of the corresponding band for the Y468F and Y475F mutants was also diminished compared to that of the wild-type control, but not to the extent observed for the Y455F or Y455/468/475F triple unphosphorylatable mutants. These findings indicate that WIP can be phosphorylated at multiple tyrosine residues and led us to a discovery programme using mass spectroscopy.

A tryptic digest of pervanadate-treated THP-1 cell lysate expressing WIP(WT)–EGFP was purified by GFP-TRAP-A beads, prepared and analysed with mass spectrometry under the conditions described in the Materials and Methods. The WIP–EGFP fusion protein sequence was identified with 73 unique peptides, accounting for 88% of sequence coverage: among them five unique phosphopeptides were also detected. All phosphopeptides identified were referred to the sequence of ‘WAS/WASL-interacting protein family member 1’ (WIPF1; UniProt code O43516) and reported in Table 1. Each phosphorylation site of WIP was manually validated taking into account results from Mascot and Andromeda. Phosphorylation of residues S234, S342, Y468 and Y455 are not reported in Uniprot, Phosida or Phospho.elm phosphorylation site databases. In our study, two out of three tyrosine residues present in the WIP sequence were found to be phosphorylated. The part of the sequence containing Y475 is included in a tryptic peptide too small to be detected in the conditions chosen for the analysis. However, a peptide comprising residues 474–481 containing one trypsin miscleavage on K478 was detected, but there was no conclusive evidence for the existence of the phosphorylated form of this peptide. These results show that cellular WIP is phosphorylated on Y455 and Y468, which can be readily detected *in situ* by mass spectrometry.

**Table 1.**
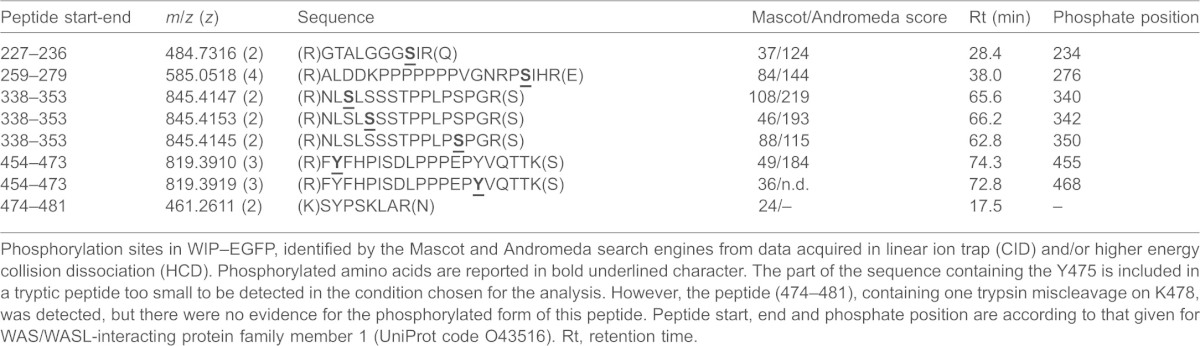
Identification of two tyrosine phosphorylation sites within the WBD of human WIP

Phosphorylation sites in WIP–EGFP, identified by the Mascot and Andromeda search engines from data acquired in linear ion trap (CID) and/or higher energy collision dissociation (HCD). Phosphorylated amino acids are reported in bold underlined character. The part of the sequence containing the Y475 is included in a tryptic peptide too small to be detected in the condition chosen for the analysis. However, the peptide (474–481), containing one trypsin miscleavage on K478, was detected, but there were no evidence for the phosphorylated form of this peptide. Peptide start, end and phosphate position are according to that given for WAS/WASL-interacting protein family member 1 (UniProt code O43516). Rt, retention time.

### WIP tyrosine mutants deficient in WASP binding confer defects in both podosome assembly and function in THP-1 cells

To determine the potential effects of tyrosine phosphorylation of WIP on WASP binding, tyrosine unphosphorylatable and phosphomimetic variants of WIP–EGFP were immunoprecipitated from THP-1 lysates and the amount of associated WASP then determined by western blotting. The unphosphorylatable WIP variants Y455F, Y468F and Y475F, as well as the triple unphosphorylatable variant Y455/468/475F all bound strongly to WASP and could immunoprecipitate WASP to a similar extent as the wild-type protein (Fig. 1D, left). Cells expressing the WIP(ΔWBD)–EGFP mutant provided a negative control, demonstrating that in the absence of the WBD of WIP, WASP is undetectable in immunoprecipitates. In contrast to the findings for the unphosphorylatable mutants, WASP was undetectable in the immunoprecipitates of cells expressing an EGFP-tagged triple phosphomimetic mutant WIP Y455E/Y468E/Y475E (Y455/468/475E) and in trace amounts in cells expressing the WIP(Y468E)–EGFP mutant, indicating that these mutations in WIP disrupt WASP binding (Fig. 1D, right). Consistent with these findings, endogenous WASP levels were increased in cells overexpressing WIP, but not in cells expressing the Y455/468/475E or Y468E phosphomimetic mutants or the WASP-binding deficient truncation mutant WIP(ΔWBD)–EGFP or EGFP control, indicating that these phosphomimetic mutants were less efficient at conferring a protective effect from proteolytic degradation on endogenous WASP.

In order to determine the role of WIP phosphorylation in podosome formation, we used a TGFβ1-dependent differentiation of THP-1 cells to macrophages model as previously described (Monypenny et al., 2011) (supplementary material Fig. S1; supplementary material Movie 1). Lentiviral-mediated delivery of short hairpin RNA (shRNA) was used to generate stable WIP-depleted THP-1 cell lines (supplementary material Fig. S2). Of five separate WIP-specific shRNA lentiviral vectors assessed in this study, two were found to reduce WIP levels significantly in THP-1 cells (supplementary material Fig. S2A). The subsequent selection of THP-1 shRNA-expressing clones that demonstrated extremely efficient WIP knockdown (relative expression ∼20% of control) allowed the generation of stable THP-1 cell lines in which endogenous WIP levels were severely depleted (supplementary material Fig. S2A, right panel). As shown in other myeloid cells, loss of WIP expression resulted in a significant reduction in both the number of cells forming podosomes (supplementary material Fig. S2B) as well as the number of podosomes observed per cell (supplementary material Fig. S2C). Moreover, when a very few ‘podosome-like’ structures were observed in knockdown cells they were small and far less distinct than those of control cells (supplementary material Fig. S2C) and were unable to degrade extracellular matrix (supplementary material Fig. S2D,E). Expression of a knockdown-resistant WIP(WT)–EGFP variant in WIP-knockdown THP-1 cells resulted in the restoration of podosomes in these cells, confirming that loss of podosome formation could be directly attributed to loss of endogenous WIP expression (compare supplementary material Movies 2 and 3).

**Fig. 2.**
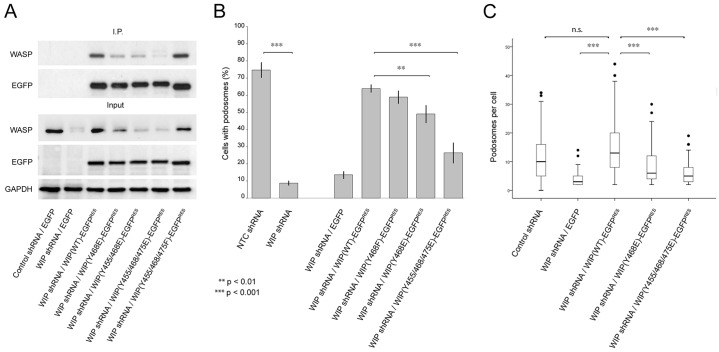
**Rescue of endogenous WASP and subsequent podosome formation by WIP phophomutant variants.** (A) Western blots demonstrating the amount of endogenous WASP immunoprecipitated by knockdown-resistant WIP–EGFP control and phosphomimetic and unphosphorylatable variants re-expressed in WIP-knockdown THP-1 cells. (B) Percentage of control and WIP-knockdown THP-1 forming podosomes following re-expression of knockdown-resistant WIP–EGFP control and phosphomimetic variants. NTC, control shRNA. Data are mean±s.e.m. (C) Box-and-whisker plots summarising podosome counts for those cells found to form podosomes in B. The box represents the 25th–75th percentiles, and the median is indicated. The whiskers show the 10th–90th percentiles. Outliers are also indicated. **P<0.01; ***P<0.001; n.s., not significant (Student's *t*-test).

**Fig. 3.**
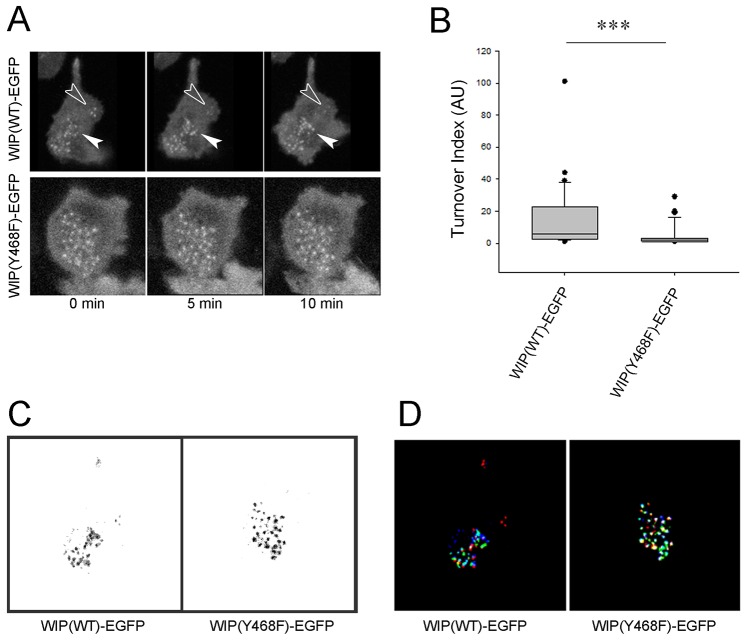
**Inhibition of WIP tyrosine phosphorylation increases podosome stability in THP-1.** (A) Expression of WIP(Y468F)–EGFP results in increased podosome stability in THP-1 when compared to wild-type control. The white arrows in the upper panel highlights a region of new podosome formation over time in a THP-1 cell expressing WIP(WT)–EGFP, whereas the empty arrow highlights a region simultaneously undergoing podosome dissassembly. Podosomes in a WIP(Y468F)–EGFP-expresing cell (lower panel) remain more stable over time. (B) Box-and-whisker diagrams of podosome adhesion turnover index. The box represents the 25th–75th percentiles, and the median is indicated. The whiskers show the 10th–90th percentiles. Outliers are also indicated. ***P<0.005 (Mann–Whitney test). (C) Composites for analysis of overall turnover of adhesion. Areas of light grey colour pixels represent dynamic adhesions whereas areas of dark grey and black colour pixels represent increasingly stable adhesions. (D) Overlap using our pseudocolour method for analysis of adhesion turnover of the series of three images taken 5 min apart shown in A. Adhesions in red represent adhesions that broke down after the first frame of the timecourse, whereas green or blue adhesions that were spatially distinct from red adhesions would have formed in the second and third frame, respectively. Stable adhesions without spatial translocation are shown in white or pink. All images are presented at the same scale as those in Fig. 4.

In addition to the wild-type protein, knockdown-resistant variants of the Y468E, Y455/468E, Y455/468/475E and Y455/468/475F WIP phosphomutants [WIP(WT)–EGFP^RES^, WIP(Y468E)–EGFP^RES^, WIP(Y455/468E)–EGFP^RES^, WIP(Y455/468/475E)–EGFP^RES^ and WIP(Y455/468/475F)–EGFP^RES^, respectively] were also generated and expressed in WIP-knockdown THP-1 cells through lentiviral-mediated gene delivery. The ability of the wild-type protein and the different mutants to both bind and restore endogenous WASP levels in THP-1 cells was then assessed (Fig. 2A). Knockdown-resistant wild-type protein and EGFP alone provided positive and negative controls, respectively. Rescue experiments revealed that although knockdown-resistant wild-type protein and the Y455/468/475F unphosphorylatable WIP mutant were capable of both binding and restoring endogenous WASP to a level similar to that found for control cells, the single Y468E, double Y455/468E and triple Y455/468/475E phosphomimetic WIP mutants were all defective in WASP binding as well as their ability to restore endogenous WASP levels. We also studied podosome dynamics in cells expressing wild-type WIP or WIP Y468F as a cell readout for the ability of unphosphorylatable WIP mutants to retain WASP binding (Fig. 2A,B). Both cell types generated podosomes as predicted from data in Fig. 2, but their behaviour was markedly different. Fig. 3A and supplementary material Movies 4 and 5 show that the WIP Y468F cells appear to have markedly more podosomes than cells expressing wild-type WIP. We quantified this from live-cell movies as described in the Materials and Methods to show that the lifetime of individual podosomes is extended in WIP Y468F cells (Fig. 3B–D), an observation we confirmed with the triple unphosphorylatable mutant (not shown). Thus a direct correlation is seen between mutation-enforced retention of WIP-WASP binding and an extended stability of podosome lifetimes.

Interestingly the triple Y455/468/475E mutant was less efficient in binding WASP than double Y455/468E indicating that the phosphorylation of the Y475 residue influences WIP–WASP interaction and might have an endogenous role even though the mass spectrometry data was unable to confirm phosphorylation of this residue (Fig. 2A; Table 1). Subsequent experiments focused on the single Y468E and triple Y455/468/475E phosphomimetic mutants as they demonstrated the most dramatic effects.

**Fig. 4.**
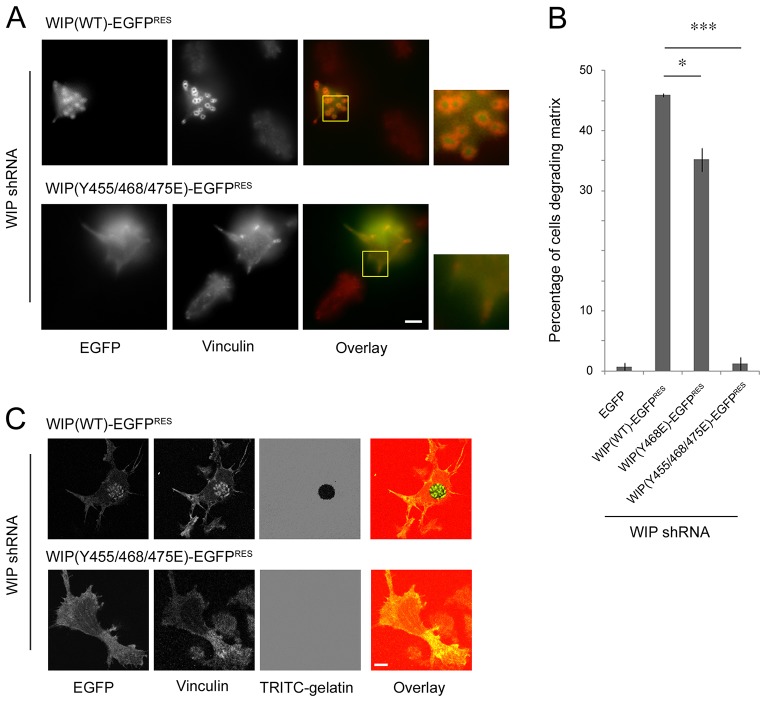
**Evaluation of the ability of wild-type and phosphomutant forms of WIP to rescue podosome formation and function in WIP-knockdown cells.** (A) Widefield images of fixed WIP-knockdown THP-1 cells re-expressing either knockdown-resistant WIP(WT)–EGFP or the triple phosphomimetic mutant WIP (Y455/468/475E)–EGFP. Additional vinculin staining demonstrates podosome rescue in cells re-expressing wild-type WIP but not the triple tyrosine phosphomimetic mutant. Insets (yellow boxes) are enlarged to highlight vinculin rings of podosomes with WIP at the core in the case of wild-type protein expression, and focal-adhesion-like structures lacking any significant WIP colocalisation in the case of triple tyrosine phosphomimetic mutant expression. (B) Statistical evaluation of the percentage of WIP-knockdown THP-1 cells degrading a TRITC-labelled gelatin matrix following expression of indicated constructs. Data are mean±s.e.m. *P<0.05, ***P<0.001. (C) Representative confocal images from gelatin degradation assays used for the statistical analysis summarised in B. Gelatin assays were performed to evaluate the ability of WIP-knockdown THP-1 cells to degrade the underlying TRITC-labelled gelatin matrix following re-expression of the indicated knockdown-resistant constructs. Scale bars: 5 µm.

Further rescue experiments revealed that phosphomimetic mutants were defective in their ability to restore podosomes in THP-1 cells (Fig. 2B,C). Both the numbers of cells forming podosomes as well as the number of podosomes observed per cell were significantly reduced in WIP-knockdown cells expressing the triple and single (Y468E) phosphomimetic tyrosine mutants when compared to WIP-knockdown cells expressing the wild-type protein (Fig. 2B,C). The Y455/468/475E triple mutant exhibited a greater defect in its ability to restore podosomes than the single Y468E mutant, fitting with the residual WASP-binding ability of this mutant (Fig. 1D). This difference was even more pronounced when the matrix-degrading potential of cells expressing these mutants was observed. Quantitative analysis of matrix degradation revealed that although WIP-knockdown cells expressing the single Y468E phosphomimetic mutant did exhibit a significantly impaired ability to degrade matrix, cells expressing the Y455/468/475E triple phosphomimetic mutant were completely devoid of any matrix-degrading function (Fig. 4A–C).

### Kinases that regulate tyrosine phosphorylation of WIP

**Fig. 5.**
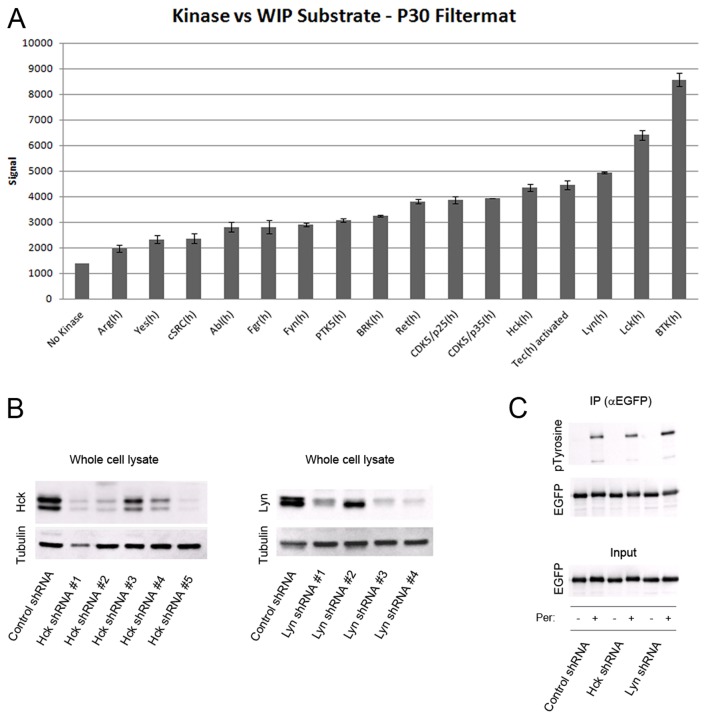
**Tec family kinases involved in WIP phosphorylation.** (A) An *in vitro* kinase assay using His_6_–WIP (human, full length) was performed by Merck Millipore following their standard protocols for the KinaseProfiler™. The histogram presents the kinase screen data. A number of kinases tested were shown to have some degree of signal above background indicative of phosphorylation of WIP. The highest responses were measured for the Tec family kinase Btk followed by Lyn and Lck. Assays were performed in duplicate with acid blanks as a negative control. Data are mean±s.d. (B) Evaluation of the efficacy of five non-overlapping Hck and Lyn shRNAs. THP-1 cells were transduced with lentivirus, subjected to puromycin selection, and finally assayed for endogenous Hck and Lyn expression. β-tubulin was used as a loading control. (C) Immunoprecipitation (IP) was performed in Hck- and Lyn-depleted cells overexpressing WIP–EGFP using an anti-GFP antibody to assess the level of WIP phosphorylation. Membranes were blotted with the anti-phosphorylated tyrosine (pTyrosine, 4G10) antibody and anti-EGFP antibody.

To identify putative kinase(s) responsible for WIP tyrosine phosphorylation, Merck Millipore's kinase profiler service was requested to perform an *in vitro* screen of candidate tyrosine kinases using baculovirus-expressed recombinant His-tagged human WIP as the substrate (Fig. 5A). Although a number of kinases tested demonstrated some degree of signal above background, significant phosphorylation activity was observed for Lck, Hck, Lyn (all members of the Src family) and for Btk, a Tec family kinase. Although Lck (lymphocyte-specific protein tyrosine kinase) is not expressed in THP-1 and other monocytic cells (also confirmed for the THP-1 cells used in this study; data not shown), both Lyn and Hck are highly expressed in THP-1 cells (our data; not shown). Thus, Btk and these two Src family kinases represent the most likely candidates for regulating WIP tyrosine phosphorylation *in situ*. To further investigate selectivity of tyrosine kinase action on WIP, we performed knockdown studies on THP-1 cells. Both Hck and Lyn levels were successfully reduced below 15% of control levels in single knockdown clones (Fig. 5B). These cells were able to proliferate and divide without apparent defect and, when induced by TGFβ1, they assembled podosomes normally (data not shown). In addition, western blot analyses showed quite distinctly that despite kinase knockdown, the levels of measured WIP tyrosine phosphorylation were undiminished compared to controls (Fig. 5C). Thus knockdown of either Hck or Lyn had no appreciable phenotype. In contrast, several attempts to generate cells from Btk-knockdown cultures failed; cells did not proliferate and cell death rapidly ensued. The lethality of the Btk knockdown renders the approach unavailable for measuring the degree of WIP phosphorylation by western blotting.

**Fig. 6.**
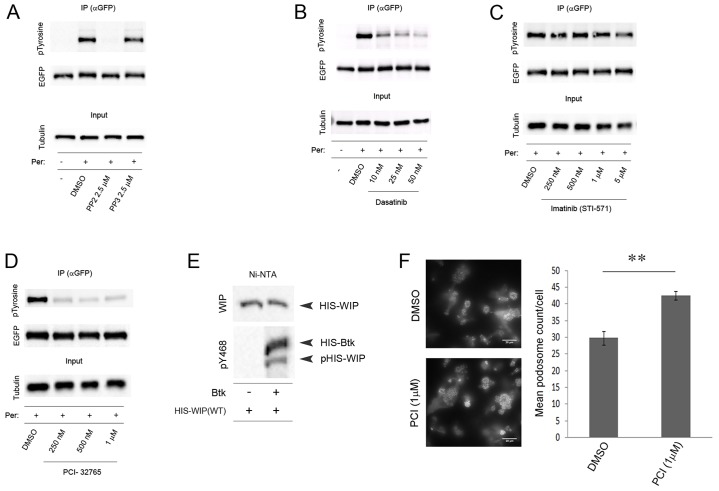
**Identification of Btk as a potent kinase for WIP tyrosine phosphorylation.** Inhibition of WIP–EGFP tyrosine phosphorylation. Total protein lysates were prepared from THP-1 cells that were stably transfected with wild-type WIP–EGFP and treated with either (A) PP2 or PP3, (B) Dasatinib, (C) STI-571 or (D) PCI-32765 at the concentration specified on the blots for 2 h before pervanadate treatment for 30 min. In all cases WIP–EGFP was immunoprecipitated (IP) from cell lysates using anti-EGFP antibody and membranes subsequently blotted with the anti-phosphorylated tyrosine (pTyrosine, 4G10) antibody, anti-EGFP antibody and anti-β-tubulin antibody. (E) *In vitro* kinase assay. Western blot analysis of recombinant His–WIP that was pre-incubated with resin and then treated with or without recombinant His-tagged active Btk. Membranes were blotted with WIP antibody and pY468 polyclonal antibody. Postions of His-tagged WIP, Btk and phosphorylated WIP are indicated on the right of the panels. (F) Effect of PCI treatment on podosome stability in THP-1 cells. Left, widefield fluorescence images of TGFβ-differentiated WIP(WT)–EGFP expressing THP-1 cells treated with either PCI (1 µM) or DMSO carrier control. Right, PCI-32765 (PCI) treatment increases the average podosome numbers in THP-1 when compared to control cells treated with DMSO carrier. Data are mean±s.e.m. **P<0.01 (Student's *t*-test). Scale bars: 20 µm.

We thus chose a second approach based upon pharmacological inhibitor studies. Inhibition of Src family kinases with PP2 (Fig. 6A) or the dual c-Abl/Src kinase inhibitor Dasatinib (Fig. 6B) blocked WIP phosphorylation. In contrast treatment of cells with PP3 (a PP2 analogue used as a negative control for PP2) or Imatinib (STI-571), an inhibitor of Abl kinase, which was not identified as a candidate kinase from the Merck *in vitro* screen, had no effect on the tyrosine phosphorylation of WIP (Fig. 6A,C). These data suggested a role for Lyn and/or Hck while excluding Btk, as it is not a Src kinase. However, a literature search identified recent findings that PP2 is rather more promiscuous than generally appreciated and also acts as a good inhibitor of Btk (Brandvold et al., 2012). Similarly, although Dasatinib primarily blocks c-Abl and Src kinases, it can also block Btk (Hantschel et al., 2008). To test the action of Btk directly, THP-1 cells expressing WIP(WT)–EGFP were treated with pervanadate and either DMSO control or increasing concentrations of the highly specific Btk inhibitor PCI-32765 (Ponader et al., 2012) before cell lysis and subsequent immunoprecipitation of WIP–EGFP to assess tyrosine phosphorylation. Btk inhibition had an apparently total inhibitory effect on the tyrosine phosphorylation of WIP at all pharmacologically relevant (Ponader et al., 2012; Tai et al., 2012) concentrations of PCI-32765 tested, demonstrating that this kinase is a key mediator of WIP phosphorylation in these cells (Fig. 6D). To validate the inhibitor data, we employed an antibody against phosphorylated Y468 to confirm phosphorylation by Btk on purified His-tagged wild-type WIP. A clear signal was observed upon addition of active Btk (Fig. 6E). An anomalous band was also seen above the WIP signal, which we identify as originating from the His–Btk itself (supplementary material Fig. S3). When we performed a similar analysis on cell-derived WIP we found that WIP–GFP, but not GFP alone or the GFP-tagged triple unphosphorylatable WIP mutant Y455/468/475F, could be phosphorylated by Btk (supplementary material Fig. S4). Finally, we studied the effect of PCI-32765 on THP-1 cells expressing wild-type WIP treated in the standard manner to generate podosomes (Fig. 6F). Podosome formation was not grossly affected but the numbers of podosomes per cell increased after PCI-32765 treatment. These data correlate with a sustained functioning of WIP–WASP complexes in the absence of phosphorylation-mediated dissociation induced by Btk.

## DISCUSSION

Until now there has been little evidence for a mechanism to explain the necessary separation of WIP from WASP that is required to facilitate WASP degradation (Chou et al., 2006; de la Fuente et al., 2007; Konno et al., 2007). Phosphorylation of serine/threonine residues clustered at functional domains of WIP have been subject to some analysis but the role of these phosphosites was not clear. In particular, phosphorylation of S488 by PKCθ was originally proposed to result in dissociation of the WIP–WASP complex (Sasahara et al., 2002). However, subsequent studies in both Jurkat cells (Dong et al., 2007) and the YTS natural killer cell line (Krzewski et al., 2006) have demonstrated that WIP and WASP remain bound together following phosphorylation of this residue. We show here that altering the phosphorylation status of S488 has no measurable effect on the separation of WIP from WASP. WIP, and in particular the C-terminal domain encompassing the WBD and the S488 residue, is known to be an intrinsically disordered protein (Haba et al., 2013), so it is possible that phosphorylation of S488 would result in a subtle shifting of the association between WIP and WASP. Recent work using a triple-colour FRET methodology has demonstrated that serine phosphorylation events can modify the spatial interaction of WIP–WASP interacting domains without driving complete protein separation (Fried et al., 2014). We also screened other candidate serine/threonine residues identified *in silico* as potential phosphorylation targets (Shu et al., 2004) and in all cases found no effects on WIP–WASP association (Fig. 1).

In contrast to the negative findings on serine and threonine residues, we demonstrate for the first time tyrosine phosphorylation of WIP. Human WIP contains three tyrosine residues that are located exclusively within the WBD, within or adjacent to sequences known to be important for WIP–WASP association (Volkman et al., 2002; Zettl and Way, 2002; Peterson et al., 2007). Y455 lies between two phenylalanine residues identified as being crucial for WIP binding to the ubiquitously expressed N-WASP and thus presumably also for binding hematopoietic-specific WASP (Zettl and Way, 2002; Peterson et al., 2007). The Y468 residue lies directly downstream of the proline-rich DLPPEP motif (residues 461–467) postulated to interact directly with the EVH1 domain of N-WASP and WASP. The EVH1 domain (also called WH1 domain) is implicated in actin remodelling through its binding to proline-rich regions of its target partner proteins. The best-characterized EVH1-domain-binding partners are members of the verprolin family of proteins. These include WIP, CR16 and WICH/WIRE (WIP and CR16 homologous protein/WIP related protein) (Antón et al., 2007). Residue Y475 lies immediately upstream of the conserved PSKxxR motif (residues 476–481) believed to interact with acidic residues within the N-WASP/WASP EVH1 domain (Volkman et al., 2002). Our initial *in silico* analysis identified Y468 as a putative target for receptor tyrosine kinases and given the significance of the position of this and other tyrosine residues with respect to important subdomains within the WBD of WIP, we set out to discover whether they represented targets for phosphorylation.

Mass spectrometry demonstrated that Y455 and Y468 are phosphorylated on human WT WIP–EGFP expressed and subsequently purified from human myeloid cells. Regarding tyrosine 475, technical limitations do not allow us to confirm with enough certainty its phosphorylation state. Phosphomimetic mutations of the three tyrosine residues completely abolish the WIP–WASP interaction, while the single phosphomimetic mutant of the central Y468 residue greatly perturbs this interaction. Using our THP-1 model in which TGFβ1-induced differentiation results in cell adhesion, podosome production and associated matrix degradation (Monypenny et al., 2011), we demonstrate a role for these tyrosine phosphorylation sites in the regulation of podosome function. Knockdown of endogenous WIP in THP-1 inhibits both podosome formation and function in these cells in a manner reminiscent of the data from WIP-null mouse dendritic cells (Chou et al., 2006). Re-expression of knockdown resistant wild-type WIP results in the efficient restoration of functional podosomes. Expression of the triple tyrosine phosphomimetic WIP mutant results in only a minimal salvage of severely disorganised structures that also lack any matrix-degradation functionality. We consequently propose that tyrosine phosphorylation of WIP provides a novel mechanism for regulating WIP–WASP association, and thereby podosome turnover and function in leukocytes.

Until now, mechanisms that directly control WIP–WASP association have remained elusive. The role of WASP phosphorylation in the regulation of cytoskeletal dynamics, podosome formation, function and cell behaviour has been the focus of far greater attention in recent years and a number of phosphorylation sites have now been identified that influence WASP function and turnover. For instance, tyrosine 291 (293 in mouse) of WASP seems to regulate both protein turnover as well as its ability to induce Arp2/3-dependent actin polymerisation (Blundell et al., 2009; Macpherson et al., 2012), while constitutive phosphorylation of WASP at serines 483 and 484 appear to be required for Arp2/3 association (Cory et al., 2003). A phosphomimetic mutant of tyrosine 291 enhances WASP-mediated actin polymerisation when compared to wild-type protein in an *in vitro* bead assay, while resting levels of filamentous actin are increased in cells expressing this mutant when compared to wild-type protein. However, murine knock-in models also reveal that phosphomimetic Y293 WASP is expressed at far lower levels than wild-type protein, an observation that can be partially reversed through inhibition of calpain (Macpherson et al., 2012) and/or the proteasome (Blundell et al., 2009) suggesting that phosphorylation of this site also targets WASP for degradation. However, the phosphomimetic versions of WASP Y291 bound to WIP with the same affinity as normal WASP (Blundell et al., 2009; Macpherson et al., 2012) indicating that an unidentified mechanism regulated the full dissociation of WIP from WASP and the latter's complete degradation. Therefore an intricate mechanism seems to be in place to balance WASP activation with its subsequent degradation. WIP has previously been reported to protect WASP from degradation (Tsuboi, 2007; Reicher et al., 2012; Chou et al., 2006; de la Fuente et al., 2007), and given that we now show that tyrosine phosphomimics of WIP both disrupt WIP–WASP association and result in a depletion of endogenous WASP levels, WIP tyrosine phosphorylation provides yet another mechanism for regulating WASP-dependent cytoskeletal rearrangements. Our current and previous work suggests that WIP may complex with phosphorylated, active WASP (Tsuboi and Meerloo, 2007; de la Fuente et al., 2007; Macpherson et al., 2012; Tsuboi, 2007) enabling it to orchestrate Arp2/3-dependent actin polymerisation events while protected from the degradation machinery (Watanabe et al., 2013). In this latter case WIP tyrosine phosphorylation would therefore provide the signal for the complete release of active WASP, resulting in its subsequent breakdown through both calpain and the ubiquitin-proteasome pathways and consequently termination of Arp2/3 mediated actin filament generation. Accordingly, the rapid breakdown of podosomes required in rapidly migrating macrophages and dendritic cells (Calle et al., 2008; Macpherson et al., 2012) would be mediated through tyrosine phosphorylation of WIP.

It is well known that members of the Src family kinases, and in particular Hck, can have significant effects on leukocyte cell morphology and movements, both in two-dimensional and three-dimensional environments (Suen et al., 1999; Guiet et al., 2008; Cougoule et al., 2010). Hck can also activate WASP through phosphorylation of Y291, leading to Arp2/3 binding and actin filament assembly (Cory et al., 2002). Hck was also identified as a WIP target, but only through a screen of Hck SH3 domain ligands (Scott et al., 2002). Based on these and other reports, it would be utilitarian to expect Hck to be the key kinase regulating podosome formation through a WIP–WASP pathway. Our findings from both the Src inhibitor PP2 study and our kinase screen all pointed to this conclusion until confounded by the finding that Hck (and Lyn) knockdown cells could still generate normal podosomes and exhibited control levels of WIP tyrosine phosphorylation. Only a re-examination of the kinase screen results and the inhibitor experiments described above led us to discover that the Tec family kinase Btk appears to be the most significant kinase controlling the levels of WIP tyrosine phosphorylation. We confirmed that Btk can indeed phosphorylate Y468 of baculovirus-generated human His-tagged WIP (Fig. 6E), but that this is more difficult to show in cell extracts where WIP would be purified along with cellular WASP (supplementary material Fig. S4). We conclude that the WIP–WASP binding in our cell extract is often so strong that we cannot disturb the interaction sufficiently to allow Btk access the tyrosine residues. It might well be that earlier events that are reported to subtly re-arrange the WIP–WASP conformation (Fried et al., 2014) are required to expose Y468 for Btk action.

Btk has been shown to associate directly with and phosphorylate WASP (Guinamard et al., 1998). Btk-null mutations have been reported in mice (XID) and in humans with X-linked agammaglobulinaemia (XLA). In both cases profound deficiencies in B-cell development and function has been observed. In osteoclasts derived from human XLA patients, podosome-based ring formation, essential for effectively resorbing calcified matrices, has been shown to be impaired (Danks et al., 2011). Similarly, the same effects are observed by treating osteoclasts with pharmacological inhibitors of Btk, both *in vitro* and *in vivo* (Tai et al., 2012). Furthermore, a WIP–WASP–Btk complex has been shown to be necessary for the activation of the TLR4 signal complex in LPS-stimulated macrophages (Sakuma et al., 2012). Based on previous reports and the present data we suggest that Btk can induce WASP activation and also facilitates its subsequent inactivation through WIP phosphorylation.

**Fig. 7.**
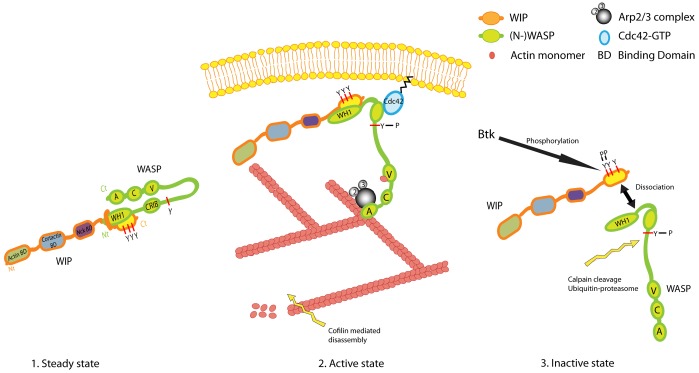
**A pathway to WASP regulation.** A model of the sequence through which WASP is rapidly activated and then inactivated in zones of podosome core assembly. WASP is initially held in an autoinhibited hairpin conformation while tightly linked to WIP. Following WASP activation through the binding of Cdc42 and other agents (Thrasher, 2002), Arp2/3 is bound to the freed C-terminal VCA domain where it facilitates actin filament assembly to initiate podosome core formation (Calle et al., 2008). Tyrosine phosphorylation of WIP within the WBD is sufficient to break the physical association of WIP and WASP, leaving WASP vulnerable to degradation through calpain (Chou et al., 2006; Macpherson et al., 2012) and/or ubiquitin pathways (Blundell et al., 2009; Reicher et al., 2012). The resulting dendritic filament array would then be vulnerable to dissolution through the action of cofilin or could be stabilised by replacement of WASP with cortactin.

Taken together, these findings demonstrate that tyrosine phosphorylation of WIP provides a new mechanism for regulating WIP–WASP dissociation and consequent degradation of WASP (Fig. 7). The general significance of these findings might also extend to WIP–N-WASP interactions that occur ubiquitously (Antón et al., 2007), to regulated cell migration (Bañón-Rodríguez et al., 2013) and, in particular, the process of invadopodia formation in some metastatic carcinomas (García et al., 2012; Yu et al., 2012).

The identification of Btk as a key mediator of WIP tyrosine phosphorylation in THP-1 cells provides important new insights into the regulatory pathways that lie upstream of WIP–WASP and consequently on podosome formation in myeloid cells and its clinical significance (Tai et al., 2012). Future work will attempt to discover the upstream signalling cascade leading to Btk activation with an emphasis on the spatial context of podosome breakdown. It would also be informative to know something of the phosphatase(s) regulating Btk in this context given that constitutive phosphorylation of WIP would greatly hinder the WASP activity necessary for podosome initiation and assembly.

## MATERIALS AND METHODS

### Cell culture

THP-1 cells (a human monocytic cell line) were purchased from the ATCC cell bank. Cells were maintained as suspension cultures in RPMI supplemented with 10% fetal calf serum (FCS). To induce podosome formation, cells were seeded in culture medium supplemented with 1 ng/ml recombinant human TGFβ1 (R&D Systems) either on coverslips or 60-mm tissue culture dishes, both coated with 10 µg/ml bovine fibronectin (Sigma). Cells were then left for 24 h to attach and form podosomes prior to subsequent experimentation.

### Generation of lentiviral vectors

cDNA encoding wild-type human WIP was amplified by PCR from pcDNA3/hWIP template plasmid and subcloned into the pCR-BLUNT vector (Invitrogen) where subsequent unphosphorylatable and phosphomimetic mutations for the relevant tyrosine, serine and threonine residues were incorporated using the QuikChange XL site-directed mutagenesis kit (Stratagene). C-terminal EGFP fusions of WIP and the various WIP mutants were generated by an intermediate subcloning step and the complete WIP–EGFP fusion construct then inserted into the multiple cloning site of the pHR'SINcPPT-SFFV (pLNT/Sffv) lentiviral transfer vector. The ΔWBD WIP truncation mutant lacking residues 450–503 (the WASP-binding domain) was generated by PCR using the same intermediate subcloning procedure. Lentiviral constructs were then sequenced and subsequently used for virus production (see below). For rescue experiments, three separate single-base silent mutations were introduced into the coding sequence of human WIP–EGFP and the associated phospho-mutants to confer resistance to shRNA-mediated knockdown following expression in THP-1 WIP-knockdown cell lines. These mutations, which reside within the human WIP sequence targeted by the Sigma Mission NM_003387.3-266s1c1 (#266) shRNA lentiviral vector, were introduced simultaneously through site-directed mutagenesis using a single primer (5′-GAAGTGCACCAATACTGGATAAGCCGAAAGGAGCTGGTGCTGGAG-3′) resulting in the following nucleotide alterations: bp 180, C to A; bp 183, A to G; and bp 186, T to G.

The mCherry–talin truncation construct was generated by PCR and subsequent subcloning as follows: PCR was used to amplify cDNA encoding residues 1975–2541 of human talin from the template plasmid (gift from Maddy Parsons, King's College London, UK) and cloned via the pCR-BLUNT vector into the MCS of the pLNT/Sffv-mCherry-MCS vector generating the mCherry–talin (1975–2541) lentiviral expression construct.

### Lentiviral gene transduction

VSV-G pseudotyped lentivirus encoding fluorescent fusion constructs or shRNAs were packaged in HEK-293T cells. Packaging cells were transiently transfected with the pCMVΔR8.91 and pMD.G accessory plasmids along with the given transfer vector: either pLNT/Sffv encoding fluorescent fusion proteins or pLKO.puro encoding shRNAs. Supernatants containing lentivirus were harvested 48 h later, 0.45 µm-filtered and stored at −80°C for future use. THP-1 cells were incubated with lentiviral supernatants in 12-well plates for 24 h and then washed thoroughly before expanding cultures. In the case of shRNA-encoding lentivirus, cells were transferred to medium supplemented with 1 µg/ml puromycin at 56 h post infection to ensure selection of only those cells that had successfully incorporated the lentiviral DNA. shRNA-encoding lentiviral vectors were from the Mission® shRNA plasmid DNA bank (Sigma). Clone IDs for WIP-specific shRNAs lentiviral plasmids used in this study were NM_003387.3-266s1c1 (#266), NM_003387.3-1388s1c1 (#1388), NM_003387.3-151s1c1 (#151), NM_003387.3-676s1c1 (#676), and NM_003387.3-1430s1c1 (#1430). Clone IDs for Hck-specific shRNAs lentiviral plasmids used were NM_002110.2-417s21c1, NM_002110.2-1735s21c1, NM_002110.2-1930s21c1, NM_002110.2-1352s21c1, NM_002110.2-1285s21c1. Clone IDs for Lyn-specific shRNAs lentiviral used were: NM_002350.2-2863s21c1, NM_002350.2-341s21c1, NM_002350.2-318s21c1, NM_002350.2-889s21c1, NM_002350.2-1570s21c1. The non-targeting control pLKO.puro plasmid (NTC) was a gift from Ester Martin (Addgene; ID 1864).

### Immunoprecipitation and western blot studies

For immunoprecipitation studies, THP-1 cells were seeded on fibronectin-coated dishes in the presence of TGFβ1 for 24 h to induce differentiation and podosome formation as described above. Cells were then lysed in 1 ml ice cold lysis buffer containing 150 mM NaCl, 1% Triton X-100, 50 mM Tris-HCl pH 7.5, 1 mM EDTA, 100 mM NaVO_4_, 50 mM NaF, and protease inhibitor mix (Roche). Lysates were clarified by centrifugation, pre-cleared for 30 min with IgG–agarose beads, and then incubated with primary antibody for 60 min at 4°C with rotation. Antibody was then captured by addition of Protein A/G agarose beads (Alpha Diagnostic) followed by an additional 60 min incubation with rotation at 4°C. Finally beads were washed once with lysis buffer and then three times with wash buffer (lysis buffer containing 500 mM NaCl) before re-suspending beads in sample buffer. In cases where tyrosine phosphorylation of immunoprecipitates was analysed, cells were pre-treated with 12 µM pervanadate for 20 min prior to lysis.

After treatment, samples for phospho-mapping analysis were incubated with GFP-TRAP-A beads (ChromoTek, Martinsried, Germany) for 2 h on a rotor at 4°C. GFP or GFP fusion proteins were pulled-down by centrifugation at 3000 ***g*** for 2 min at 4°C. Beads where then washed three times using immunoprecipitation lysis buffer before being snap frozen.

Antibodies used for immunoprecipitation and western blotting were as follows. Goat polyclonal anti-WIP (G-20) and mouse monoclonal anti-WASP (Clone B-9) were from Santa Cruz Biotechnology. Rabbit polyclonal and mouse monoclonal anti-EGFP were from MBL (#598) and Roche (#11-814-460-001), respectively. Mouse monoclonals against GAPDH (clone 6C5) and phosphotyrosine (4G10 Platinum) were from Millipore. For detection of phosphotyrosine in immunoblots, 100 mM NaVO_4_ was included during membrane blocking and primary and secondary antibody incubation stages.

For the quantification of protein bands in immunoblots, images were processed in ImageJ as follows. Images were inverted, processed to subtract background using default ImageJ settings, and the integrated density function then used to quantify protein band intensity. Protein bands were then normalised to that of their corresponding GAPDH loading controls. The level of normalised protein expression for each lane of a blot was expressed as a percentage of that for the control NTC shRNA lane.

### Live-cell imaging

Wide-field studies were conducted using an Olympus IX81 wide-field inverted microscope equipped with an environment chamber accurately maintained at 37°C. All imaging was conducted using 100×, NA 1.4 objective and excitation and emission filter wheels (Sutter) equipped with filters optimised for EGFP and mCherry epifluorescence. Image acquisition was controlled by Metamorph imaging software (Universal Imaging). In order to monitor podosome turnover time-lapse imaging was conducted over a 30-min period with fluorescence images acquired at 30-s intervals.

#### Analysis of adhesion turnover

The podosome turnover in THP-1 cells expressing EGFP constructs was performed by visualising the GFP signal using an IX71 Olympus inverted epifluorescence microscope and images were acquired every 30 s. Micrographs were processed using Adobe Photoshop® version 7.0 to threshold the podosome cores in the cells. To analyse the persistence of the spatial localisation of podosome cores, five interference reflection microscopy (IRM) images taken 5 min apart were used. Each image was thresholded to produce white adhesions on a black background and then inverted as black adhesions on a white background. Next, the black value of each image was divided by five to obtain dark gray corresponding to adhesions (i.e. 256/5 on the scale of 1–256). The images were then overlapped using the ‘difference’ function in Adobe Photoshop. We thus obtained a composite image with five relevant grey levels. The lightest grey level represented pixels that were present in one of the five images (adhesion points last for less than 5 min), and the darkest grey level represented pixels that were present in five out of five images (i.e. adhesion points last for 20 min). Therefore, the areas of lighter grey colour pixels represent dynamic adhesions whereas areas of dark grey and black colour pixels represent increasingly stable adhesions during the selected time course of measurement. Using Mathematica™ 5.2 notebooks (Macpherson et al., 2012), we could quantify the percentage of pixels corresponding to each grey level per image, which allowed us to calculate a turnover index by dividing the percentage of pixels present in one or two frames by the percentage of pixels present in four or five frames (Macpherson et al., 2012). Thus, a ratio of unstable adhesion over stable adhesion in each live cell was obtained. A higher value of the turnover index represents more dynamic cell adhesion. A Mann–Whitney test was used to assess the significance of experimental results.

### Immunocytochemistry

THP-1 cells were seeded on fibronectin-coated coverslips (10 µg/ml) in growth medium supplemented with 1 ng/ml TGFβ1 and cultured for up to 24 h. Cells were then fixed with 3.7% paraformaldehyde, permeabilised with 0.01% Triton X-100, and blocked in 3% BSA in PBS before incubation with primary antibodies. Filamentous actin was stained using Alexa-Fluor-465-conjugated phalloidin (Molecular Probes), which was included during the primary antibody incubation step. Vinculin was labelled using the mouse monoclonal clone hVIN-1 (Sigma) and Alexa-Fluor-568-conjugated donkey anti-mouse secondary antibody (Molecular Probes). Coverslips were mounted on slides using Prolong Gold (Invitrogen) and imaged using a Zeiss LSM 510 inverted confocal microscope using a 63× Plan Fluor objective.

#### Podosome counting

For quantification of cells with or without podosomes, or podosome numbers per cell, a minimum of three repeat assays were scored with a minimum of 40 cells counted in fixed preparations from each experiment. ImageJ was used to acquire counts and a Student's *t*-test performed on the resulting combined data sets. A cell was considered to contain podosomes if it contained more than one identifiable structure.

### Matrix degradation assay

#### Preparation of fluorescent gelatin

2 mg/ml of gelatin was dissolved in a solution containing 61 mM of NaCl and 50 mM sodium borohydrate (Na_2_B_4_O_7_) (pH 9.3) (Sigma-Aldrich) and then incubated at 37°C for 1 h. After dissolution, 2 mg of Rhodamine (Sigma-Aldrich) was added and mixed for 2 h in darkness. This mixture was then dialysed overnight at room temperature in PBS in complete darkness. Dialysis was repeated for 2 days with two buffer changes per day. After addition of 2% (w/v) sucrose a quick spin was performed to remove insoluble material and small aliquots were stored in the dark at 4°C.

#### Preparation of fluorescent-gelatin-coated coverslips

Prior to coating, 13-mm glass coverslips were sterilized in 70% ethanol for 30 min at room temperature and rinsed in 99% ethanol. Air-dried coverslips were then coated with warm Rhodamine-conjugated gelatin, using around 200 µl per 13-mm round coverslips to cover the surface for 5 min. Each coverslip was inverted onto a 200 µl volume of 0.5% ice-cold glutaraldehyde in PBS held on a parafilm support. After 15 min in the dark at room temperature, each coverslip was then transferred to a multi-well dish with the coated side up and gently washed three times with PBS. Coverslips were subsequently incubated with 1 ml of sodium borohydride (5 mg/ml) in PBS for 3 min at room temperature and washed three times with PBS. Finally coverslips were sterilized again in 70% ethanol for 5 min, dried for 10–15 min under a sterile hood and then quenched in complete medium for 1 h at 37°C.

#### Procedure

2×10^5^ cells were cultured on gelatin-coated coverslips and stimulated with 1 ng/ml of human recombinant TGFβ for 15–24 h, then fixed in 3.7% PFA and processed for immunofluorescence as described above. For quantification of matrix degradation, cells were imaged using a Zeiss LSM 510 inverted confocal microscope. Five random fields at a 100× (Plan fluor objective) magnification were imaged. For each experimental condition, cells exhibiting gelatin degradation immediately beneath a cell were scored. The percentage of cells showing gelatin degradation activity was calculated as an indicator of podosome functionality. The data shown are a mean of three separate experiments compared using Student's *t*-test.

### Mass spectrometry

#### Sample preparation

For phospho-mapping analysis, 10^7^ THP-1 cells overexpressing WIP–EGFP were seeded on fibronectin-coated dishes in the presence of 1 ng/ml of TGFβ for 24 h. Cells were pre-treated with 12 µM pervanadate for 20 min prior to lysis to inhibit endogenous phosphatases. To perform phosphorylation site analysis on an isolated protein, endogenous phosphatases had to be kept inactive before purification by immunoprecipitation. After treatment samples were lysed using 1 ml of the lysis buffer: 150 mM NaCl, 50 mM Tris-HCl, 1 mM EDTA, 1 mM EGTA, 10 mM sodium β-glycerophosphate, 50 mM NaF, 5 mM sodium pyrophosphate, 1 mM NaOV, 1× Triton X-100, proteinase inhibitor tablet, 0.27 M sucrose, 1 mM DTT. Lysates were incubated with 15 µl of GFP-TRAP-A beads (ChromoTek, Martinsried, Germany) for 2 h on a rotor at 4°C. GFP or GFP fusion proteins were pulled-down by centrifugation at 3000 ***g*** for 2 min at 4°C. Beads were then washed three times using immunoprecipitation lysis buffer before being snap frozen.

#### Proteolytic digestion of proteins ‘in gel’

Immunoprecipitated WIP–EGFP was eluted from the beads using 1% SDS, separated by SDS-PAGE gels and visualized with Colloidal Coomassie Blue. The band containing WIP–EGFP fusion protein was excised from the gel and digested with trypsin.

#### Liquid chromatography–mass spectrometry

The tryptic digest obtained was separated by nanoscale C18 reverse-phase liquid chromatography [EASY-nLC II (Thermo Fisher Scientific)] coupled to a Linear Trap Quadrupole (LTQ) Orbitrap Velos mass spectrometer (Thermo Fisher Scientific). Samples were loaded on a pre-column (C-18 Biosphere 5 µm, 120 Å–200 µm×0.2 cm) for desalting, and subsequently eluted, at a flow of 0.6 µl/min, into an analytical column (C-18 Biosphere 5 µm, 120 Å–100 µm×15 cm).

The eluting peptide solutions were electrosprayed into the mass spectrometer via a nanoelectrospray ion source. The mass spectrometer was operated in positive ion mode and used in data-dependent acquisition mode. A full scan (FT-MS) was acquired with resolution of 30,000 over mass range of 350–2000 amu, and the top ten most intense ions were selected for fragmentation using both available fragmentation modes: linear ion trap (CID) and higher energy collision dissociation (HCD) in two different acquisitions.

To improve the fragmentation of phosphopeptides in CID mode, the ‘multistage activation algorithm’ in the Xcalibur software was enabled. Fragmentation spectra in HCD were acquired in the FTMS analyser at a resolution of 7500 in centroid mode. Normalized collision energy used was 35 for both multistage activation CID and HCD methods. The ‘lock mass’ function (lock mass = 445.120036 Da) was enabled for MS and MS/MS HCD scan modes.

#### Data analysis

The raw data obtained from HCD and multistage activation CID acquisitions were processed and pooled with Raw2MSN and all MS/MS samples were analysed using Mascot (Matrix Science, London, UK; version 2.3.02), querying SwissProt database (release 2011_11, selected for *Homo sapiens*, 20249 entries) using the digestion enzyme trypsin and allowing for two miscleavages.

Mascot was searched with a fragment ion mass tolerance of 0.5 Da and a parent ion tolerance of 10 ppm. The iodoacetamide derivative of cysteine was specified in Mascot as a fixed modification. Oxidation of methionine and phosphorylation of serine, threonine and tyrosine were specified in Mascot as variable modifications.

Scaffold (version 3.1.2, Proteome Software Inc., Portland, OR) was used to validate MS/MS-based peptide and protein identifications. Peptide identifications were accepted if they could be established at greater than 95.0% probability as specified by the Peptide Prophet algorithm, resulting in a peptide false discovery rate (FDR) of 0.7%. Additional data processing was carried out using Maxquant/Andromeda (version 1.2.0.18). The search engine was forced to consider unlabelled peptides by setting the multiplicity value to 1. Fixed and variable modifications, enzyme and numbers of miscleavages were the same chosen previously for Mascot. The peptide, protein and site FDR was set at 0.01; peptides identified with less than six amino acid residues were not included in the results.

### Kinase profiling assay

Kinase assays were conducted using Merck Millipore's FlexLab service based on the protocols detailed at http://www.millipore.com/techpublications/tech1/pf3036.

### Inhibitor assays

THP-1 cells were seeded on fibronectin-coated 60-mm diameter dishes at a density of 5×10^6^ cells/ml and treated with 2 ng/ml of TGFβ for 15 to 24 h. Podosome-forming cells were treated for 2 h with the indicated concentrations of the following inhibitors: PP2 (Tocris Bioscience), PP3 (Tocris Bioscience), Dasatinib (Selleckchem.com) PCI-32765 (Selleckchem.com) and STI-571 (Selleckchem.com). Cells were then treated with 12 µM pervanadate for 30 min prior to lysis. Cells were washed with ice-cold PBS and lysed using 1 ml lysis buffer supplemented with sodium fluoride, sodium pyrophosphate, sodium orthovanadate and sodium glycerophosphate. Pre-cleared lysates were incubated with 15 µl of GFP-TRAP-A beads (ChromoTek, Martinsried, Germany) for 2 h on a rotor at 4°C. EGFP or EGFP fusion proteins were immunoprecipitated and beads subsequently collected by centrifugation at 3000 ***g*** for 2 min at 4°C. Beads where then washed three times using immunoprecipitation lysis buffer before loading onto polyacrylamid gels. Nitrocellulose membrane were then probed with the anti-phosphorylated-tyrosine 4G10 (Millipore), anti-β-tubulin (Sigma-Aldrich) and anti-EGFP (Roche) antibodies.

### *In vitro* Btk phosphorylation assay

Aliquots of 3 µg of purified recombinant His–WIP (Merck-Millipore) were incubated with Ni-NTA resin (Qiagen) in wash buffer (50 mM Tris-HCl pH 8.0, 150 mM NaCl, 25 mM imidazole pH 8.0, 0.1% Triton X-100) for 1 h at 4°C with rotation. The resin was washed three times in wash buffer and once in kinase buffer (25 mM MOPS pH 7.2, 12.5 mM glycerol 2-phosphate, 25 mM MgCl2, 5 mM EGTA, 0.25 Mm DTT, 100 µM ATP). The resin was then incubated with 20 µl of kinase buffer containing 100 ng of His-tagged active Btk (Sigma) for 1.5 h at 30°C. The resin was washed three times in wash buffer before boiled with FSB and subjected to western blot analysis.

For the Btk phosphorylation assay using the overexpressed EGFP-tagged proteins from THP-1 cells, immunoprecipitation was performed using GFP-TRAP-A beads. Five million THP-1 cells that contain either overexpressed EGFP, EGFP-tagged WIP (WT) or WIP(Y455/468/475F) were washed two times with PBSA at 4°C and resuspended in 0.7 ml culture cell lysis buffer (50 mM Tris-HCl pH 7.5, 150 mM NaCl, 1 mM Na_3_VO_4_, 50 mM NaF, 5% glycerol, 1% Triton X-100, 1× protease inhibitors). Cells were disrupted by vigorous pipetting and the resulting cell lysate was cleared of nuclei and membranes by centrifugation at 16,100 ***g ***for 10 min at 4°C. Clarified cell lysates were then incubated with GFP-TFAP-A beads for 1 h at 4°C with rotation. The beads were washed three times in cell lysis buffer and once in kinase buffer (25 mM MOPS pH 7.2, 12.5 mM glycerol 2-phosphate, 25 mM MgCl_2_, 5 mM EGTA, 0.25 Mm DTT, 100 µM ATP) before being incubated with 20 µl of kinase buffer containing 20 ng of His-tagged active Btk for 30 min at 30°C. The beads were then washed three times in cell lysis buffer before boiled with final sample buffer (FSB; 60 mM Tris-HCl pH 6.8, 2% SDS, 10% glycerol, 5% β-mercaptoethanol, 0.01% Bromophenol Blue and 1 mM Na_3_VO_4_) and subjected to western blot analysis.

The pY468 rabbit polyclonal antibody that was used in these assays was generated against the peptide PEP[pY]VQTTKSYPSKC of human WIP, a service provided by GenScript.

## Supplementary Material

Supplementary Material
