# Supplementary material for: Tyrosine phosphorylation of WIP releases bound WASP and impairs podosome assembly in macrophages
Source: J Cell Sci. 2015 Jan 15;128(2):251–65. doi: 10.1242/jcs.154880 (PMC4294773; doi:10.1242/jcs.154880)
Supplement: Supplementary Material [file supp_128_2_251__index.html]

Tyrosine phosphorylation of WIP releases bound WASP and impairs podosome assembly in macrophages — Supplementary Material 

# Tyrosine phosphorylation of WIP releases bound WASP and impairs podosome assembly in macrophages

## JCS154880 Supplementary Material

**Files in this Data Supplement:**

- **Supplementary Material**
