# Supplementary material for: Tyrosine phosphorylation of WIP releases bound WASP and impairs podosome assembly in macrophages
Source: J Cell Sci. 2015 Jan 15;128(2):251–65. doi: 10.1242/jcs.154880 (PMC4294773; doi:10.1242/jcs.154880)
Supplement: Supplementary Material [file supp_128.2.251_JCS154880.pdf]

Figure S1

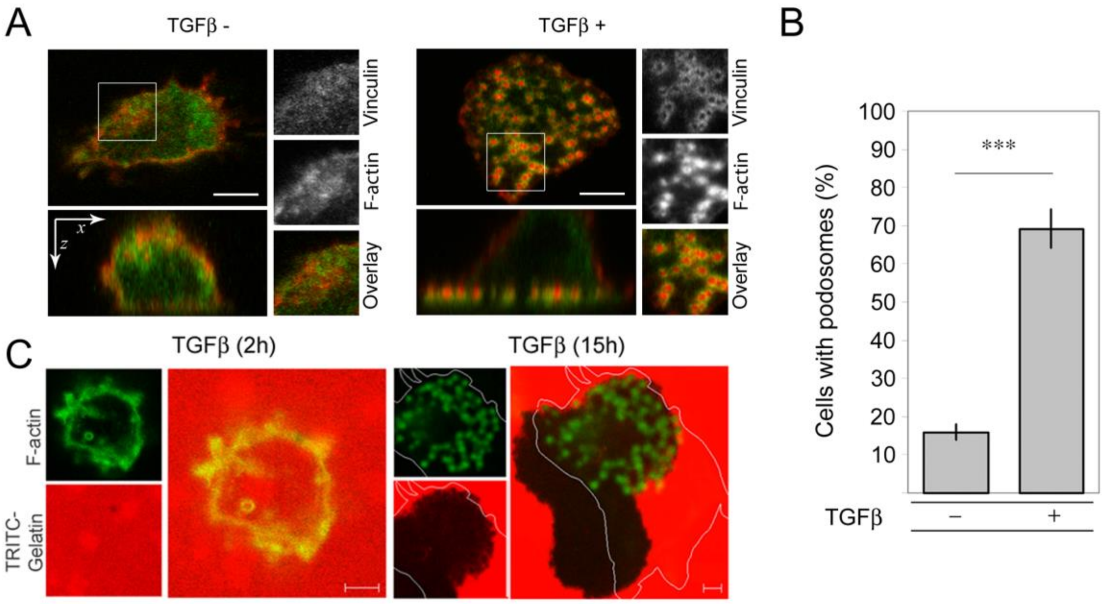
A
TGFβ -
TGFβ +
Vinculin
F-actin
Overlay
x
z
B
100
90
80
70
60
50
40
30
20
10
Cells with podosomes (%)
***
TGFβ
–
+
C
TGFβ (2h)
TGFβ (15h)
F-actin
TRITC-Gelatin

Figure S2

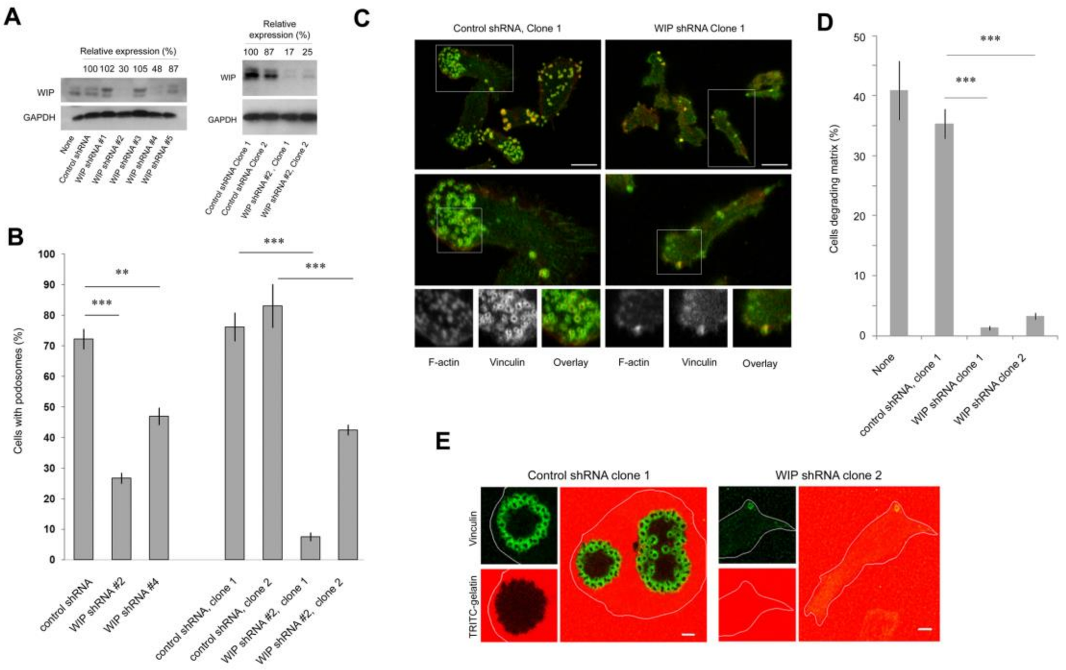
A
Relative expression (%)
100 102 30 105 48 87
WIP
GAPDH
None
Control shRNA
WIP shRNA #1
WIP shRNA #2
WIP shRNA #3
WIP shRNA #4
WIP shRNA #5
Relative expression (%)
100 87 17 25
Control shRNA Clone 1
Control shRNA Clone 2
WIP shRNA #2, Clone 1
WIP shRNA #2, Clone 2
B
Cells with podosomes (%)
control shRNA
WIP shRNA #2
WIP shRNA #4
control shRNA, clone 1
control shRNA, clone 2
WIP shRNA #2, clone 1
WIP shRNA #2, clone 2
C
Control shRNA, Clone 1
WIP shRNA Clone 1
F-actin
Vinculin
Overlay
D
Cells degrading matrix (%)
None
control shRNA, clone 1
WIP shRNA clone 1
WIP shRNA clone 2
E
Control shRNA clone 1
WIP shRNA clone 2
Vinculin
TRITC-gelatin

Figure S3

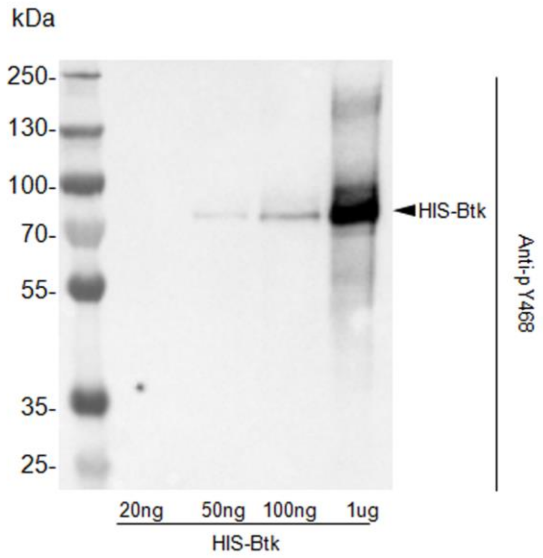
kDa
250-
130-
100-
70-
55-
35-
25-
HIS-Btk
Anti-p Y468
20ng
50ng
100ng
1ug
HIS-Btk

Figure S4

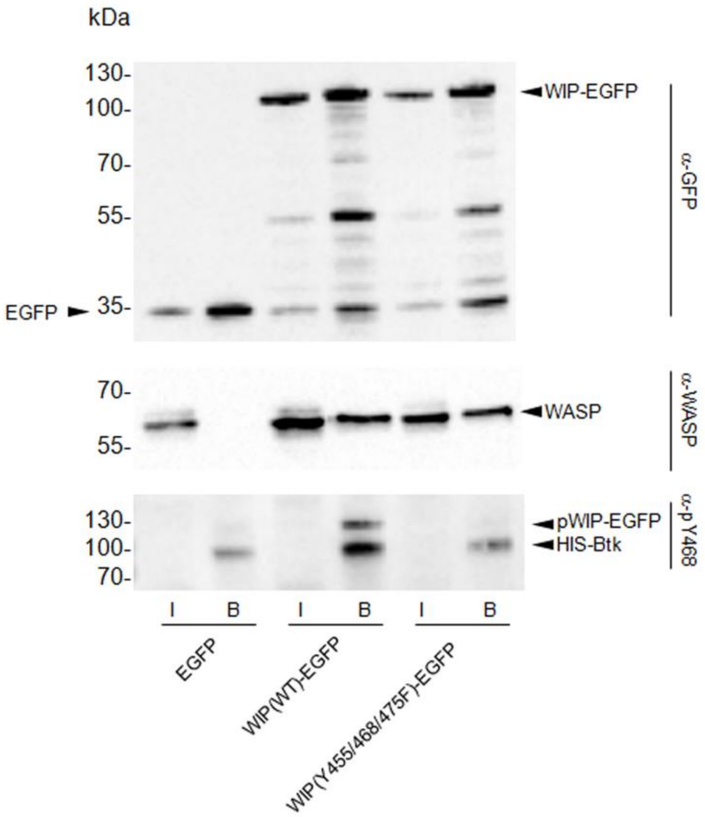

kDa
130-
100-
70-
55-
35-
EGFP
WIP-EGFP
α-GFP
70-
55-
WASP
α-WASP
130-
100-
70-
pWIP-EGFP
HIS-Btk
α-pY468
I
B
I
B
I
B
EGFP
WIP(WT)-EGFP
WIP(Y455/468/475F)-EGFP

JOCES/2014/154880: Figure legends for Supplementary files

Supplementary figure 1. TGFβ1-dependent differentiation of THP-1 as a model for the study of the leukocyte podosome. (A) THP-1 seeded on fibronectin substrate in the presence of 1 ng/ml TGFβ1 form multiple podosomes following 24h in culture. Immunocytochemical analysis reveals the presence of multiple podosomes, as determined by filamentous actin and vinculin staining, in only those cells cultured in the presence of TGFβ1. Although cells are able to attach to the underlying substrate in the absence of TGFβ1 podosomes are rarely observed. (B) Statistical evaluation of the proportion of THP-1 forming podosomes following culture in the absence or presence of TGFβ1 (***, $p < 0.001$, Student's t-test). (C) Ability of THP-1 to degrade an underlying gelatin matrix demonstrates that TGFβ-dependent THP-1 podosomes are functional. The red channel represents rhodamine-conjugated gelatin and the green channel alexa-488 conjugated phalloidin. Scale bar =5 μm.

Supplementary figure 2. shRNA-mediated knockdown of WIP inhibits podosome formation. (A) Evaluation of the efficacy of five non-overlapping WIP shRNAs. THP-1 were transduced with lentivirus, subjected to puromycin selection, and finally assayed for endogenous WIP expression (left hand blot). Clonal cell lines were generated from those cell populations infected with the non-targeting control and WIP shRNA #2 lentivirus to generate control and WIP-deficient cell lines respectively (right-hand blot). At the top of each blot the level of endogenous WIP expression for each WIP shRNA expressing cell lines is expressed as a percentage of that found for the NTC control. (B) Statistical evaluation of the number of cells producing podosomes from THP-1 cell populations harbouring the given control and WIP-specific shRNAs. (**, $p < 0.01$; ***, $p < 0.001$, Student's t-test). (C) Representative confocal images demonstrating the presence and absence of intact podosomes in control and

WIP-knockdown cells respectively. Scale bar: 10 μm. Lower panels represent enlargements of the white outlined regions in corresponding panels above. (D) Statistical evaluation of the ability of control and WIP-shRNA expressing THP-1 cell lines to degrade an underlying gelatin matrix. (***, $p < 0.001$). (E) Representative confocal images of control shRNA and WIP shRNA-expressing THP-1 cells cultured on a TRITC-gelatin matrix. Scale bar: 2 μm. In each case the white line traces the border of each cell. Cells were fixed and subsequently subjected to immunostaining with an anti-vinculin antibody (green), TRITC-gelatin is in red.

Supplementary figure 3. HIS-Btk is recognised by pY468 antibody. Western blot analysis of recombinant HIS-Btk using pY468 antibody. The amounts of HIS-Btk that was incubated with resin are indicated at the bottom of the blot. HIS-Btk is indicated on the right of the blot.

Supplementary figure 4. Overexpressed WIP(WT)-EGFP but not WIP(Y455/468/457F)-EGFP can be phosphorylated by Btk. WIP (WT) and WIP (Y455/468/457F) with an C-terminal EGFP tag were overexpressed in THP-1 cells. The EGFP-tagged proteins were immunoprecipitated from cell lysates using GFP-TRAP-A beads. The precipitated proteins were treated with recombinant HIS-tagged active Btk and analysed by western blotting with antibodies against EGFP, WASP and pY468. To estimate the amounts of overexpressed proteins, 10% of each lysate was used as input. EGFP-tagged proteins, input (I) and bound (B) are indicated at the bottom of the panels. Endogenous WASP, HIS-tagged Btk, EGFP-tagged WIP and phosphorylated WIP are indicated on the right of the panels.

Supplementary movie #1. live-cell imaging: Visualisation of podosome turnover in migrating THP-1. Multi-channel time-lapse image sequence of a THP-1 cell co-expressing WIP (WT)-EGFP and mCherry-Talin (1975-2541). In the channel overlay panel on the right

mCherry-Talin is pseudocoloured green and WIP (WT)-EGFP pseudocoloured red. A sharpness filter was applied to both the WIP (WT)-EGFP and mCherry-Talin channels before combining to produce the pseudocoloured overlay. The single channel panels represent the original, unsharpened image data. Images were acquired every 30s over a 60 min observation period using a 100x magnification objective as described in the methods.

Supplementary movie #2. live-cell imaging: Loss of podosome assembly in WIP-KD THP-1. Multi-channel time-lapse image sequence of a WIP-knockdown THP-1 cell co-expressing EGFP and mCherry-Talin (1975-2541). In the channel overlay panel on the right mCherry-Talin is pseudocoloured green and EGFP pseudocoloured red. As in the previous movie a sharpness filter was applied to both the EGFP and mCherry-Talin channels before combining to produce the pseudocoloured overlay. The single channel panels represent the original, unsharpened image data. Images were acquired every 30s over a 30 min observation period using a 100x magnification objective as described in the methods.

Supplementary movie #3. live-cell imaging: Podosomes restoration in WIP-KD THP-1 following expression of KD-resistant WIP (WT)-EGFP. Multi-channel time-lapse image sequence of a WIP-knockdown THP-1 cell co-expressing knockdown-resistant WIP (WT)-EGFP and mCherry-Talin (1975-2541). In the channel overlay panel on the right mCherry-Talin is pseudocoloured green and WIP (WT)-EGFP pseudocoloured red. As in the previous movies a sharpness filter was applied to both the WIP (WT)-EGFP and mCherry-Talin channels before combining to produce the pseudocoloured overlay. Again, the single channel panels represent the original, unsharpened image data. Images were acquired every 30s over a 30 min observation period using a 100x magnification objective as described in the methods.

Supplementary movie #4. live-cell imaging: Podosome turnover in THP-1 expressing WIP(WT)-EGFP. Single-channel time-lapse image sequence of a THP-1 cell expressing WIP(WT)-EGFP. Images were acquired every 30s over a 20 min observation period using a 100x magnification objective as described in the methods.

Supplementary movie #5. live-cell imaging: Podosome turnover in THP-1 expressing WIP(Y468F)-EGFP. Single-channel time-lapse image sequence of a THP-1 cell expressing WIP(Y468F)-EGFP. Images were acquired every 30s over a 20 min observation period using a 100x magnification objective as described in the methods.

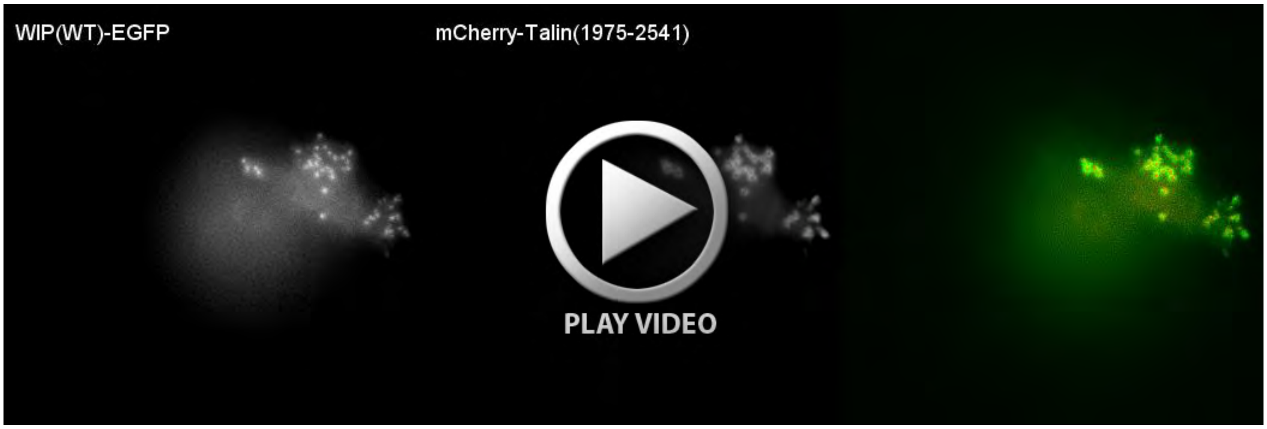

Movie 1.

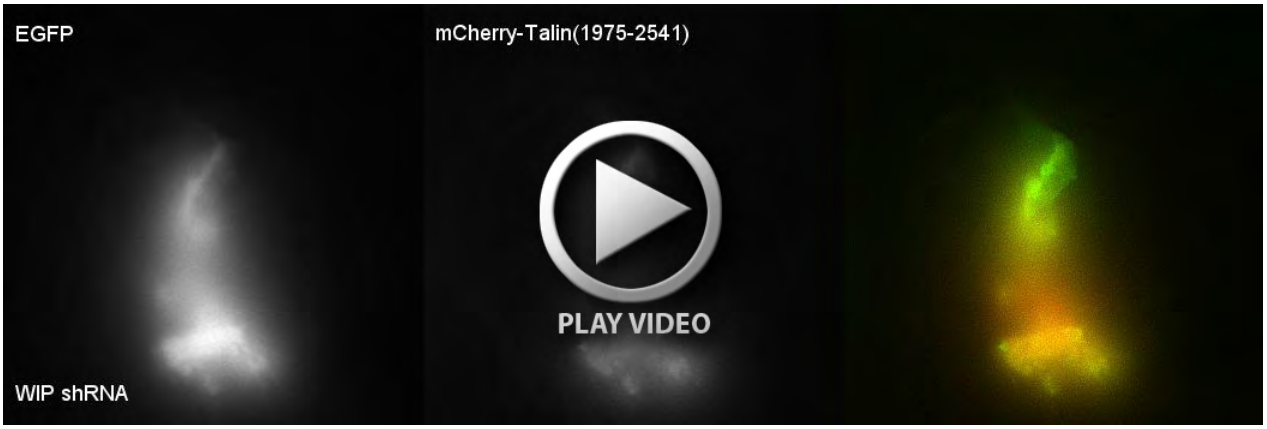

Movie 2.

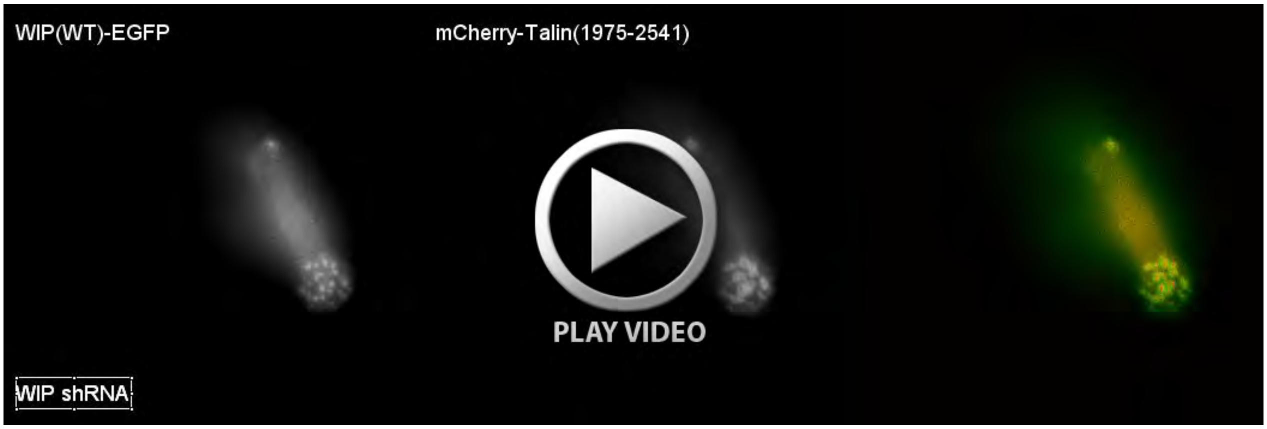

Movie 3.

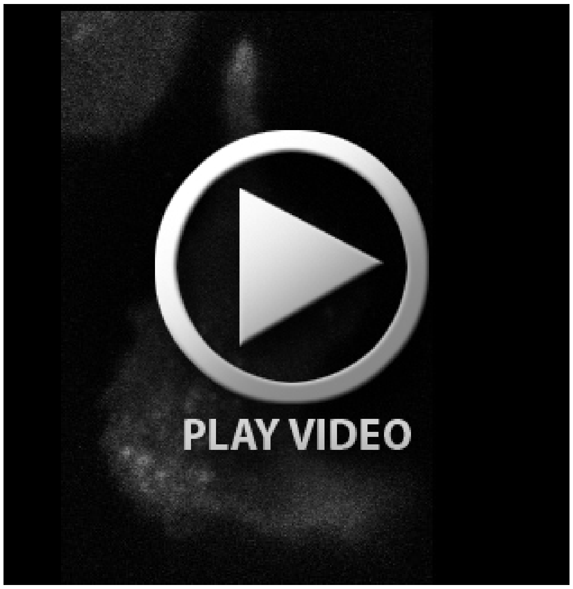

Movie 4.

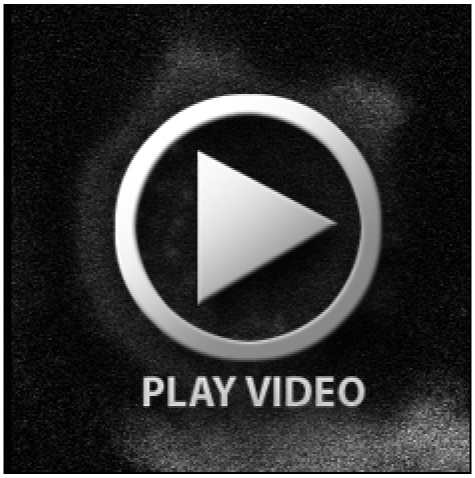

Movie 5.
